# Maternal BMI as a predictor of methylation of obesity-related genes in saliva samples from preschool-age Hispanic children at-risk for obesity

**DOI:** 10.1186/s12864-016-3473-9

**Published:** 2017-01-09

**Authors:** Kathryn Tully Oelsner, Yan Guo, Sophie Bao-Chieu To, Amy L. Non, Shari L. Barkin

**Affiliations:** 1 College of Medicine, Medical University of South Carolina, 96 Jonathan Lucas St, Suite 601, MSC 617, Charleston, SC 29425 USA; 2 Center for Quantitative Research, School of Medicine, Vanderbilt University, 2220 Pierce Ave, 571 Preston Research Building, Nashville, TN USA; 3 Department of Biological Sciences, Vanderbilt University, 1210 BSB, 465 21st Ave S, Nashville, TN USA; 4 Department of Anthropology, University of California San Diego, 9500 Gilman Drive, La Jolla, CA 92093 USA; 5 Department of Pediatrics, Vanderbilt University School of Medicine, 2200 Children’s Way, Doctor’s Office Tower 8232, Nashville, TN 37232-9225 USA; 6 Pediatric Obesity Research, Diabetes Research and Training Center, Vanderbilt University School of Medicine, 2200 Children’s Way, Doctor’s Office Tower 8232, Nashville, TN 37232-9225 USA

**Keywords:** Obesity, Hispanic children, Epigenetics, Methylation, Methionine, Cysteine biosynthesis, Homocysteine

## Abstract

**Background:**

The study of epigenetic processes and mechanisms present a dynamic approach to assess complex individual variation in obesity susceptibility. However, few studies have examined epigenetic patterns in preschool-age children at-risk for obesity despite the relevance of this developmental stage to trajectories of weight gain. We hypothesized that salivary DNA methylation patterns of key obesogenic genes in Hispanic children would 1) correlate with maternal BMI and 2) allow for identification of pathways associated with children at-risk for obesity.

**Results:**

Genome-wide DNA methylation was conducted on 92 saliva samples collected from Hispanic preschool children using the Infinium Illumina HumanMethylation 450 K BeadChip (Illumina, San Diego, CA, USA), which interrogates >484,000 CpG sites associated with ~24,000 genes. The analysis was limited to 936 genes that have been associated with obesity in a prior GWAS Study.

Child DNA methylation at 17 CpG sites was found to be significantly associated with maternal BMI, with increased methylation at 12 CpG sites and decreased methylation at 5 CpG sites. Pathway analysis revealed methylation at these sites related to homocysteine and methionine degradation as well as cysteine biosynthesis and circadian rhythm. Furthermore, eight of the 17 CpG sites reside in genes (*FSTL1, SORCS2, NRF1, DLC1, PPARGC1B, CHN2, NXPH1*) that have prior known associations with obesity, diabetes, and the insulin pathway.

**Conclusions:**

Our study confirms that saliva is a practical human tissue to obtain in community settings and in pediatric populations. These salivary findings indicate potential epigenetic differences in Hispanic preschool children at risk for pediatric obesity. Identifying early biomarkers and understanding pathways that are epigenetically regulated during this critical stage of child development may present an opportunity for prevention or early intervention for addressing childhood obesity.

**Trial registration:**

The clinical trial protocol is available at ClinicalTrials.gov (NCT01316653). Registered 3 March 2011

## Background

Prevalence of childhood obesity remains a significant public health concern, especially in Hispanic populations who have the higher pediatric obesity rates [1]. Despite having increased risk of developing pediatric and adult obesity compared to other ethnic groups [2], Hispanic children are currently underrepresented in public health research. This is particularly significant as Hispanics are the most populous and rapidly growing ethnic minority in the United States, meaning this population’s health comorbidities secondary to obesity will increase healthcare costs and rates of morbidity [3, 4]. Genetic predisposition, exposure to unhealthy dietary options, and lack of adequate physical activity have all been identified as contributors to pediatric obesity [5]. However, recent literature indicates a more nuanced dynamic mechanism associated with later childhood and adult obesity that reflects the interaction between genetics, environment, and developmental stage via epigenetic modifications [6–8]. While the study of the epigenome is complex, it has the potential to inform the prevention and treatment of pediatric obesity by enhancing our understanding of timing and the mechanisms by which the genetic code could be susceptible to environmental influences [9, 10].

Genetic factors are known to affect multiple cellular and metabolic pathways underlying the development of obesity such as: adipogenesis and fat storage, adipocyte accumulation, the hypothalamic-pituitary adrenal (HPA) system stress response affecting cardiovascular and metabolic health, gastrointestinal tract regulatory signals, orexigenic and anorexigenic and satiety mechanisms, and insulin regulation [11, 12]. For example, there is evidence that adipocyte growth in number and size is established early, by the age of 2, and is indicative of future weight trajectory [13]. Additionally, maternal Body Mass Index (BMI) is correlated with child’s BMI status at age 6 [14] and is a better indicator of child’s BMI trajectory than child birth weight alone [6]. Current maternal BMI has been shown to be significantly associated with current child’s BMI more than other maternal socioeconomic factors (including age, marital status, education). These and other studies indicate that both current and pre-pregnancy maternal BMI are significantly associated with child’s BMI trajectory [15, 16].

However, the epigenetic mechanisms affecting potential candidate genes linked to biological processes, such as adipocyte accumulation, are relatively unknown in pediatric populations. Epigenetic mechanisms regulate the level of gene transcription, which occurs through multiple processes including DNA methylation [8, 17]. Research indicates that methyl groups can bind the genetic code in either a heritably stable or an environmentally-induced transient manner, affecting the child’s trajectory for excessive weight gain relative to height [18, 19]. There is some evidence of *in utero* environmentally-induced methylation associated with exposure to maternal gestational diabetes [20, 21]; maternal inadequate nutrition or insulin resistance that can cause an adaptive response in the child, resulting in epigenetic modifications signaling caloric retention [22–25]. In addition, Liu and colleagues reported that maternal pre-pregnancy BMI was associated with alterations in offspring DNA methylation in cord blood at CpG sites annotated to genes related to the development of various complex chronic diseases, such as cardiovascular disease [9].

While the study by Liu et al. linked maternal weight phenotypes (normal weight; overweight; and obese) to epigenetic patterns in offspring neonatal cord blood samples [9], children between the ages 3–5 have been relatively understudied in the field of epigenetics. This is likely due to the convenience of neonatal cord blood at a younger age and the limited feasibility of obtaining blood samples until older ages. Yet, this age range is particularly important as it falls closest to the adiposity rebound stage and could play a significant role in a child’s future BMI trajectory [26]. Thus, examining the link between current maternal BMI and young children’s DNA methylation patterns, particularly among Hispanic children at high risk for obesity, can fill important gaps in current epigenetic research.

Saliva is a promising yet relatively underutilized source of DNA [27, 28]. Previous studies indicate that up to 74% of DNA in saliva comes from white blood cells, although there is high variability in individual samples [29]. Additionally, saliva is part of the gastrointestinal tract, and therefore, an important tissue to examine in obesity research [30]. Furthermore, using saliva samples rather than blood to yield epigenetic information introduces a more practical method to measure epigenetics from young children in a variety of settings, including the home and community [31].

While epigenetic patterns are tissue-dependent and results may not be consistent with other tissues [32], this study examines if there is variation in salivary DNA methylation in young children at risk for later obesity. We had three study aims: 1) to examine the association of maternal BMI phenotype with methylation patterns in preschool Hispanic child saliva by analyzing CpG sites located in genes previously associated with obesity [33]; 2) to assess if preschool child saliva would yield distinct epigenetic signatures in children at-risk for obesity compared to children of normal weight mothers; and 3) to identify biological pathways and genes in children correlated with maternal BMI. These findings could then identify potential epigenetic signatures in saliva among young children at risk for obesity, but not yet obese.

## Methods

### Ethics statement

The study was approved by the Vanderbilt University Institutional Review Board (IRB No. 120643). Data were collected after a parent/legal guardian signed a written informed consent, for themselves and their child, in their preferred language (English or Spanish). The clinical trial protocol is available at ClinicalTrials.gov (NCT01316653). Registered 3 March 2011. The data for this manuscript derive from baseline salivary samples obtained prior to randomization.

### Sample population study subjects

This study involved baseline saliva samples from 92 Hispanic parent-preschool children dyads, who are participating in an ongoing randomized controlled trial (RCT), the Growing Right Onto Wellness (GROW) Trial [34]. Children were not necessarily firstborn. Eligibility criteria for the RCT included: child 3–5 years old; child’s BMI ≥50 and <95% (at risk for obesity, but not yet obese) [35]; parental commitment to participate in a 3-year randomized controlled trial; parent age ≥18 years; parent and child in good health, without medical conditions necessitating limited physical activity as evaluated by a pre-screen; dyad considered underserved as indicated by the parent self-reporting if they or someone in their household participated in programs such as TennCare (Medicaid), CoverKids, Special Supplemental Nutrition Program for Women, Infants, and Children (WIC), Food Stamps, and/or free and reduced price school meal. These children are considered to be at high risk for later childhood and adult obesity [36].

### Phenotypic data

Height and weight were measured in accordance with standard anthropometric measurement procedures [37, 38]. Both values were collected twice, with the mean of the two closest measures used as the final measurement. BMI was calculated as weight in kilograms divided by the square of height in meters. Table 1 outlines the phenotypic and demographic data for the sample population.

**Table 1:** Sample demographics

Child Age, mean (SD)	3.78 (0.78)
Age 3, No. (%)	40 (43)
Age 4, No. (%)	32 (35)
Age 5, No. (%)	20 (22)
Maternal Age, mean (SD)	31.70 (5.75)
Gender, No. (%)	
Female	46 (50)
Male	46 (50)
Maternal BMI (kg/m^2), mean (SD)	29.80 (7.60)
Maternal Waist Circumference (cm), mean (SD)	98.16 (16.04)
Child BMI (kg/m^2), mean (SD)	16.80 (0.83)
Child Waist Circumference (cm), mean (SD)	53.34 (3.17)
Child and Parent Race, No. (%)	
Hispanic/Hispanic	92 (100)

### Procedures

All salivary samples were collected at baseline in the GROW RCT, before any interventions occurred, using the Oragene DNA saliva kit following a strict protocol [39]. All study members wore gloves and immediately capped specimen after collection. Samples were sent to the Vanderbilt genetic core for assessment of quality and quantity prior to storage in the Vanderbilt Technologies for Advanced Genomics (VANTAGE) core at Vanderbilt University. DNA extraction was performed as per DNA Genotek’s recommendations using the PrepIT L2P reagent. Extracted DNA was stored in individually barcoded cryovials at −80° Fahrenheit. For children, saliva was obtained using the “baby brush” approach, in which small sponges attached to plastic handles are inserted between cheek and gumline to absorb saliva [40]. The phenotypic data derived from a baseline survey and objectively measured anthropomorphic data was collected from participating mother-child pairs.

### Identification of CpG Probes

We focused our analysis on 11,387 CpG sites that resided in 936 genes that have been previously reported in genome-wide association studies (GWAS) to have association with childhood obesity in a Hispanic population [41]. Moreover, the original GWAS study’s initial sample size was 815 Hispanic children from 263 families [41].

### Assay method

Genome-wide DNA methylation was conducted on the 92 saliva samples using the Infinium Illumina HumanMethylation 450 K BeadChip (Illumina, San Diego, CA, USA), which interrogates >484,000 CpG sites associated with ~24,000 genes [42]. This microarray spans 99% of genes in the Reference Sequence database, with an average of 17 CpG sites per gene region, and has been previously validated for consistency [43]. Arrays were processed using standard protocol [44], with three samples randomly selected to serve as duplicates and one sample run with HapMap DNA to test functionality of reagents. Duplicates were measured for high technique consistency with Pearson correlation coefficient (>.99).

### Quality control

Methylation data were quality controlled using Illumina GenomeStudio (V2011.1), Methylation module (V1.9.0). The data processing and quality control were performed using the Illumina GenomeStudio, methylation module 1.8. The GenomeStudio had built in protocols for conducting methylation array normalization. We utilized Background Subtraction, where the background value is derived by averaging the signals of built-in negative control bead types. Outliers are removed using the median absolute deviation method. Background normalization is capable of minimizing the amount of variation in background signals between arrays. This is accomplished using the signals of built-in negative controls, which are designed to be thermodynamically equivalent to the regular probes but lack a specific target in the transcriptome. Negative controls allow for estimating the expected signal level in the absence of hybridization to a specific target. The average signal of the negative controls is subtracted from the probe signals. As a result, the expected signal for unexpressed targets is equal to zero. Samples with lower than 98% call rate (i.e. <485,000 probes) were excluded.

Any non-specific cross-reacting probes, probes carrying common SNPs (MAF >1%), or any probes with p-values greater than 0.05 for more than 20% of the sample were sequentially excluded [45, 46]. One saliva sample was removed after quality control analysis (total analytic sample of *n* = 91). Normalization at CpG island level was performed using internal control subtracting background noise.

### Statistical analysis

We employed an advanced statistical method called elastic net in order to select a reduced set of CpG markers for regression analyses because the number of CpG markers is substantially greater than the number of subjects [47]. The elastic net method provides variable selection to produce parsimonious and interpretable models without being severely limited by the sample size [47, 48]. While multiple test corrections are not necessary for elastic net [49–51], to demonstrate the more common presentation of results, we report Hochberg adjusted p-values [52].

The elastic net was performed prior to linear regression analysis to identify CpG sites associated with maternal BMI, due to its clinical relevance to childhood obesity for the Hispanic population [53–55]. The CpG sites selected by elastic net were then used in a univariate model to examine the individual association with maternal BMI using a linear regression model where the main outcome was child CpG methylation and the main predictor was maternal BMI, adjusting for covariates that included: child BMI, maternal age, child gender, and child age.

### Pathway analysis

Functional analysis of differentially methylated genes was conducted using Ingenuity Pathway Analysis (IPA) on child CpG Sites determined to be significantly methylated and associated with maternal BMI by linear regression. The analysis utilized the Ingenuity Knowledge Base (QIAGEN), a structured collection of five million findings from biomedical literature and integrated third party databases that contains 40,000 nodes with 1,480,000 edges representing cause-effect relationships. These cause-and-effect relationships take into account expression, transcription, activation, molecular modification, transport, and binding [56].

## Results

### Methylation analysis

**Fig. 1:**
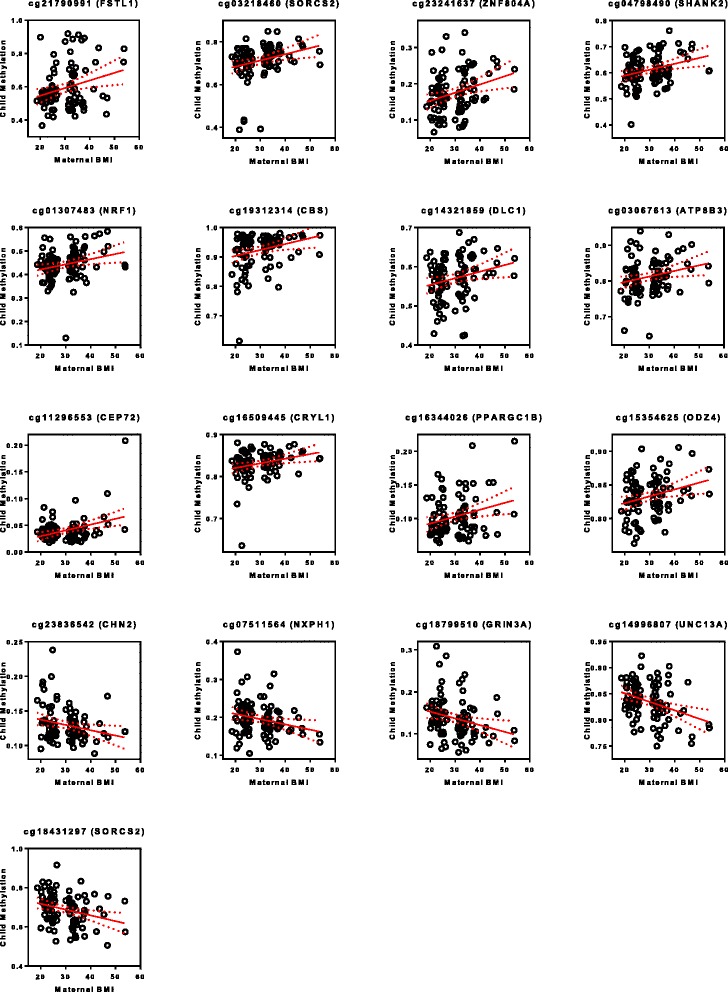
Direct Correlation of CpG Site with Maternal Obesity. Maternal BMI per Child methylation level for the 17 CpG sites with significant methylation as determined by linear regression. Genes indicated in parenthesis; Red line indicating linear regression; dotted line indicating 95% confidence interval

The elastic net identified 17 CpG sites that were associated with maternal BMI (Fig. 1). Twelve of the 17 CpGs had increased methylation with increased maternal BMI while 5 of the 17 CpGs had decreased methylation associated with increased maternal BMI. Of these CpGs, all were significantly and independently associated with maternal BMI, as determined by linear regression (Table 2). Five CpGs were found within an enhancer region of the associated gene, 1 CpG was associated with the promotor region, and 5 CpGs were unclassified.

**Table 2:** Linear regression analysis of child DNA methylation signal predicted by maternal BMI

Probe	Gene	Effect	Standard Deviation	*P*-Value^*^	Associated Pathology
**cg21790991** ^a^	***FSTL1***	**0.005058**	**0.001776**	0.0156	**Obesity** [65, 66]
**Cg03218460**	***SORCS2***	**0.003025**	**0.001091**	0.0165	**Cardiovascular disease** [67] **SORCS family associated with Type 1 and 2 Diabetes** [67–70]
Cg23241637	*ZNF804A*	0.002443	0.00081	0.0143	Schizophrenia [71–75]
Cg04798490	*SHANK2*	0.002391	0.000787	0.0143	Autism [76–81]
**cg01307483**	***NRF1***	**0.002344**	**0.000879**	0.0172	**Type 2 Diabetes** [82–85]
cg19312314	*CBS*	0.001952	0.000813	0.0243	Cardiovascular Disease [86] Homocystinuria [87–89]
**cg14321859**	***DLC1***	**0.001801**	**0.00076**	0.0243	**Insulin-Pathway** [90] **Hepatocellular Carcinoma** [91–94]
cg03067613	*ATP8B3*	0.001547	0.000672	0.0253	Reproduction [95, 96] Liver Disease [97]
cg11296553	*CEP72*	0.001159	0.000312	0.0041	Ulcerative colitis [86]
cg16509445	*CRYL1*	0.001091	0.000427	0.0204	Hepatocellular Carcinoma [98, 99]
**Cg16344026**	***PPARGC1B***	**0.001021**	**0.000403**	0.0204	**Obesity** [100–102]
Cg15354625	*ODZ4*	0.000932	0.000402	0.0253	Bipolar Disorder [103–105]
**cg23836542**	***CHN2***	**−0.00081**	**0.000337**	0.0243	**Insulin-Pathway** [106] **Type 2 Diabetes** [107, 108]
**cg07511564**	***NXPH1***	**−0.00135**	**0.00061**	0.0295	**Type 2 Diabetes** [109, 110]
cg18799510	*GRIN3A*	−0.00171	0.000633	0.0172	Schizophrenia [111, 112]
Cg14996807	*UNC13A*	−0.00174	0.00048	0.0041	Amyotrophic lateral sclerosis [113–116]
**Cg18431297**	***SORCS2***	**−0.00316**	**0.001079**	0.0146	**Cardiovascular disease** [67] **SORCS family associated with Type 1 and 2 Diabetes** [67–70]

*Hochberg adjusted *p*-values noted

^a^Bolded CpG sites are associated with Obesity, Diabetes, and/or the Insulin-Pathway

### Pathway analysis

The top 10 canonical signaling pathways included cysteine biosynthesis, homocysteine degradation, cysteine biosynthesesis III, superpathway of methionine degradation, D-glucuronate degradation I, and Circadium Rhythm Signaling (Table 3).

**Table 3:** Top 10 signaling pathways derived from top differentially methylated genes

Top Canonical Pathways	
	Adjusted *P*-value
Cysteine Biosynthesis/Homocysteine Degradation	1.55E-03
D-glucuronate Degradation I	2.33E-03
Cysteine Biosynthesis III (Mammalia)	1.46E-02
Superpathway of Methionine Degradation	2.45E-02
Circadian Rhythm Signaling	2.53E-02
Top Diseases and Bio Functions	
Developmental Disorders (3 Molecules)	4.70E-02 - 7.76E-04 3
Hematological Disease (6 Molecules)	3.98E-02 - 7.76E-04 6
Hereditary Disorder (5 Molecules)	4.70E-02 - 7.76E-04 5
Metabolic Disease (5 Molecules)	3.96E-02 - 7.76E-04 5
Neurological Disease (8 Molecules)	4.41E-02 - 7.76E-04

## Discussion

To our knowledge, this is the first study examining DNA methylation in saliva samples obtained from preschool-age Hispanic children to investigate epigenetic patterns in children at-risk for later childhood obesity. We identified 17 CpG sites in saliva of children to be associated with maternal BMI, indicating a potential intergenerational transmission of risk for obesity in children of obese mothers.

### Effect of maternal BMI phenotype on child DNA methylation in saliva

The 17 CpG probes identified by the Elastic net analysis from saliva samples are consistent with Comuzzie et al’s original GWAS study of whole blood DNA samples from 815 Hispanic children in 263 families [41]. The 17 CpG sites in Table 2 were independently and significantly associated with maternal BMI. While child age was controlled for in the linear regression analysis, these patterns may change as children age. We plan to assess epigenetic signatures and the development of childhood obesity over time in future research.

### Top differentially methylated genes

Eight out of the 17 CpG sites selected by the elastic net analysis reside in genes that are associated with obesity, diabetes, or the insulin pathway by prior studies (Table 2). Specifically, the PPARGC1B and NXPH1 genes have been associated with childhood obesity in Brazil and with diabetes in the Mexican-Mestizo populations respectively. Since our population was solely Hispanic, we found it interesting that the saliva epigenetic signatures were consistent with known genetic causes of obesity and diabetes in similar populations of Hispanic origins. There are similarities between obesogenic and oncogenic states, namely cellular proliferation and inflammation, and it is interesting to note, that in this study two of the genes with significant methylation, DLC1 and CRYL1, are associated with hepatocellular carcinoma. These genes have biologic plausibility of contributing to an increased risk of childhood obesity. However, because the saliva samples were derived from children within similar non-obese BMI ranges, these significant differences may indicate changes occurring in numerous different pathways even before the clinical presentation of obesity.

One unexpected finding, was that 5 of the 17 CpG sites with significant methylation have strong associations with neurological disease in the literature, specifically schizophrenia, bipolar disorder, autism, and amyotrophic lateral sclerosis (Table 2) [57, 58].

### Pathway analysis

Pathway analysis identified significant enrichment of genes within pathways involved in conical signaling with respect to methylation in cysteine biosynthesis, homocysteine degradation, cysteine biosynthesis III, and methionine degradation pathways (Table 3). These pathways have been associated with obesity previously. For example, cysteine biosynthesis has been found to be positively associated with risk of obesity in Hispanic children [59], and total plasma cysteine has been independently associated with obesity and insulin resistance in the same population [60]. Furthermore, homocysteine degradation has been found to be positively associated with morbidly obese patients [61], and restriction of methionine intake has been shown to have a significant increase in fat oxidation [62]. Circadian rhythm was also identified as a top canonical pathway. Circadian rhythms regulate many biological processes and cellular metabolic pathways. Disruption of circadian rhythm has an adverse effect on metabolic function [63].

### Salivary vs blood assays

Comuzzie et al. used DNA samples from whole blood in a GWAS study to identify novel genetic loci associated with the pathophysiology of childhood obesity in Hispanic children ages 4–19 years old [41]. Using these same genes in our analyses of saliva, we identified 17 CpG sites in a Hispanic pediatric population with significant methylation and associated with maternal obesogenic phenotypes. Thus, this proof of principle study demonstrated that saliva is a probable viable medium for epigenetic testing, which in this case, was consistent to whole blood findings, but we acknowledge that further testing would have to include both blood and saliva samples from the same Hispanic pediatric population to holistically assess the similarities between these 2 tissue samples. Previous studies that have investigated both of these tissues in other patient populations indicate that saliva and whole blood findings are consistent, so we would anticipate further investigations to yield similar findings [25, 26].

### Limitations

Although prior literature indicates that DNA methylation levels in saliva are similar to those in peripheral blood, skin fibroblasts, and buccal swab DNA, it may not reflect the epigenome of adipose tissue, muscle, pancreas, GI system, and pituitary [64], which are implicated in the development of obesity. Furthermore, we acknowledge that we cannot assess whether these findings would be consistent in blood samples in this specific population, although prior literature seems to indicate that we would find similar epigenetic methylation patterns if tested. We did not correlate gene expression data with methylation changes, and thus can only speculate on the implications for a child’s BMI trajectory. P-adjusted values were less likely to be significant due to the large number of genes analyzed in the Illumina Human Methylation Bead Chip 450 K, making our statistically significant findings important, but likely under-identifying other potential statistically significant differential methylation patterns.

## Conclusions

Results of this proof of principle study indicate that saliva is a practical way to obtain biologically plausible findings in an epigenetic analysis of preschool-age children. It is important to understand the potential pathways that could be epigenetically regulated in preschool aged children who are not currently obese but at higher risk of obesity. Moreover, saliva, an easily accessible tissue, could assist in the future identification of early biomarkers of later childhood obesity and metabolic dysfunction, presenting an opportunity for prevention or early intervention for addressing childhood obesity.

## Abbreviations

BMI: Body Mass Index
GROW: The Growing Right Onto Wellness Trial
GWAS: Genome-wide Association Studies
HPA: Hypothalamic-Pituitary Adrenal
RCT: Randomized Controlled Trial
VANTAGE: the Vanderbilt Technologies for Advanced Genomics
WIC: The Special Supplemental Nutrition Program for Women, Infants, and Children
